# 1-(2,6-Dihydroxy-4-methoxyphenyl)-2-(4-hydroxyphenyl) Ethanone-Induced Cell Cycle Arrest in G_1_/G_0_ in HT-29 Cells Human Colon Adenocarcinoma Cells

**DOI:** 10.3390/ijms15010468

**Published:** 2014-01-02

**Authors:** Ma Ma Lay, Saiful Anuar Karsani, Sri Nurestri Abd Malek

**Affiliations:** 1 Institute of Biological Sciences, Faculty of Science, University of Malaya, Kuala Lumpur 50603, Malaysia; E-Mails: saiful72@um.edu.my (S.A.K.); srimalek@um.edu.my (S.N.A.M.); 2 University of Malaya Centre for Proteomics Research (UMCPR), University of Malaya, Kuala Lumpur 50603, Malaysia

**Keywords:** cell cycle, annexin V, MTT cell proliferation, *Phaleria macrocarpa* (Scheff.) Boerl, 1-(2,6-dihydroxy-4-methoxyphenyl)-2-(4-hydroxyphenyl) ethanone (DMHE), western blot

## Abstract

1-(2,6-Dihydroxy-4-methoxyphenyl)-2-(4-hydroxyphenyl) ethanone (DMHE) was isolated from the ethyl acetate fraction of *Phaleria macrocarpa* (Scheff.) Boerl fruits and the structure confirmed by GC-MS (gas chromatography-mass spectrometry) and NMR (nuclear magnetic resonance) analysis. This compound was tested on the HT-29 human colon adenocarcinoma cell line using MTT (method of transcriptional and translational) cell proliferation assay. The results of MTT assay showed that DMHE exhibited good cytotoxic effect on HT-29 cells in a dose- and time-dependent manner but no cytotoxic effect on the MRC-5 cell line after 72 h incubation. Morphological features of apoptotic cells upon treatment by DMHE, e.g., cell shrinkage and membrane blebbing, were examined by an inverted and phase microscope. Other features, such as chromatin condension and nuclear fragmentation were studied using acridine orange and propidium iodide staining under the fluorescence microscope. Future evidence of apoptosis/necrosis was provided by result fromannexin V-FITC/PI (fluorescein-isothiocyanate/propidium iodide) staining revealed the percentage of early apoptotic, late apoptotic, necrotic and live cells in a dose- and time-dependent manner using flow cytometry. Cell cycle analysis showed G_0_/G_1_ arrest in a time-dependent manner. A western blot analysis indicated that cell death might be associated with the up-regulation of the pro-apoptotic proteins Bax PUMA. However, the anit-apotptic proteins Bcl-2, Bcl-xL, and Mcl-1 were also found to increase in a time-dependent manner. The expression of the pro-apoptotic protein Bak was not observed.

## 1. Introduction

The pharmacological and/or biological activity of organic molecules isolated from animals, plants, or microbes can be used to treat human diseases. Humans have relied on natural products as a source of medicine for thousands of years. Traditional medicine systems in countries such as Egypt, China and India were formed based on plant-based natural products [1]. A recent study by the World Health Organization (WHO, Geneva, Switzerland) showed that 80% of the world’s population is still heavily reliant on traditional medicine [2]. Natural products may have one or more biological activity that may include antioxidant activity, antimicrobial activity, antibacterial activity, antifungal activity, anti-cancer activity, anti-hypertension, anti-diabetes activity and many more.

*Phaleria macrocarpa* (Scheff.) Boerl belongs to the Thymelaeceae family and is known as Mahkota Dewa in Indonesia. All parts of the *P. macrocarpa* plant, namely the fruits, seeds, leaves and stem contain beneficial chemical components that may potentially be developed into drugs. There has been some research on the components of the *P. macrocarpa* fruit with regard to its biological activity. For instance, the butanol fraction of the fruits has been found to significantly prevent an alloxan-induced diabetic state by enhancing hepatic antioxidant activity in treated animals [3]. The pericarp and mesocarp from the *P. macrocarpa* fruit have been shown to possess antioxidant and anti-inflammatory activities due to the presence of phenolic and flavonoid compounds. The pericarp, mesocarp and seed displayed cytotoxic activity in HT-29, MCF-7 and HeLa cell lines [4]. It has also been shown that the ethanol extract of the flesh from *P. macrocarpa* fruit was toxic towards the HeLa cell line [5]. Gallic acid isolated from the fruits has been shown to exhibit anticancer properties [6].

The compounds 2,6′,4-trihydroxy-4-methoxybenzophenone and 4′,6-dihydroxy-4-methoxybenzophenone 2-*O-*β-d-glucopyranoside isolated from the *P. macrocarpa* fruit showed anti-proliferative activity against the breast cancer cell line, MDA-MB231 and was also reported to possess apoptosis induction activity [7]. A compound (referred to as DLBS 1442) from the *P. macrocarpa* fruit has been shown to be effective in assuaging the effects of primary dysmenorrhoea in addition to abdominal pain and other symptoms related to premenstrual syndrome [8].

In the present study, 1-(2,6-dihydroxy-4-methoxyphenyl)-2-(4-hydroxyphenyl) ethanone (DMHE) was isolated from the ethyl acetate fraction of the *P. macrocarpa* fruits using column chromatography and its identity was confirmed by gas chromatography mass spectrometry (GC-MS) and nuclear magnetic resonance (NMR) analysis. The bioactive compound (DMHE) was screened on three cancer cell lines (HT-29, A-549, MCF-7) and the normal human fibroblast cell line (MRC-5) using MTT cell proliferation assay. The mode of cell death in HT-29 cells was then studied by observing morphological changes of the cells.

## 2. Results and Discussion

### 2.1. Isolation of 1-(2,6-Dihydroxy-4-methoxyphenyl)-2-(4-hydroxyphenyl) Ethanone (DMHE)

The ethyl acetate fraction (9.8 g) was subjected to silica gel column chromatography (400 g, Merck Kieselgel 60, 0.063–0.200 mm mesh size; Merck, Darmstadt, Germany): initial elution with hexane, followed by ethyl acetate enriched with increasing percentages of acetone, and monitoring with TLC (Thin layer chromatography), resulted in nine fractions: FF-1 (0.7 g), FF-2 (0.79 g), FF-3 (0.42 g), FF-4 (0.7 g), FF-5 (0.5 g), FF-6 (0.8 g), FF-7 (1.5 g), FF-8 (1.8 g) and FF-9 (2.3 g). These fractions were identified using GC-MS and NMR. Components of selected fractions were purified by re-crystallization and separation using a specific solvent.

**Figure 1.**
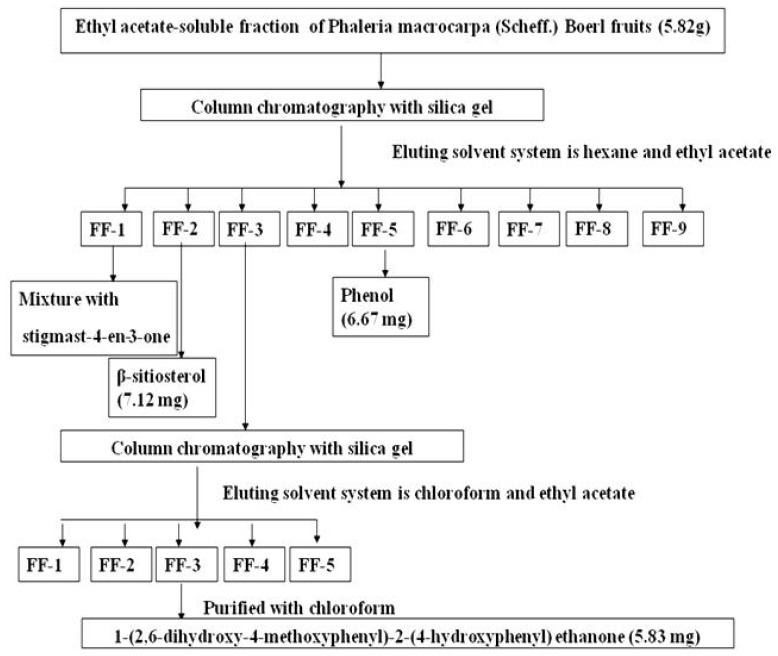
Flowing showing isolation of 1-(2,6-dihydroxy-4-methoxyphenyl)-2- (4-hydroxyphenyl) ethanone (DMHE) using silica gel column chromatography.

Our results showed a mixture with stigmast-4-en-3-one (*m*/*z* 412) was obtained from fraction one (FF-1) and β-Sitosterol (*m*/*z* 414) was purified from fraction 2 (FF-2). Fraction 3 (FF-3, 0.42 g) was further purified by silica gel column chromatography (2 × 60 cm, 200 g) by eluting with chloroform and ethyl acetate (99:1 to 99:1). This resulted in four fractions and following further separation by TLC lead to the identification of 1-(2,6-dihydroxy-4-methoxyphenyl)-2-(4-hydroxyphenyl) ethanone (DMHE, 5.83 mg; *m*/*z* 274). DMHE was then purified with chloroform and analyzed by gas chromatography-mass spectrometry (GC-MS), ^1^H NMR and ^13^C NMR. A flowchart describing the steps involved the purification of DMHE is shown in Figure 1.

### 2.2. Identification of 1-(2,6-Dihydroxy-4-methoxyphenyl)-2-(4-hydroxyphenyl) Ethanone (DMHE)

**Figure 2.**
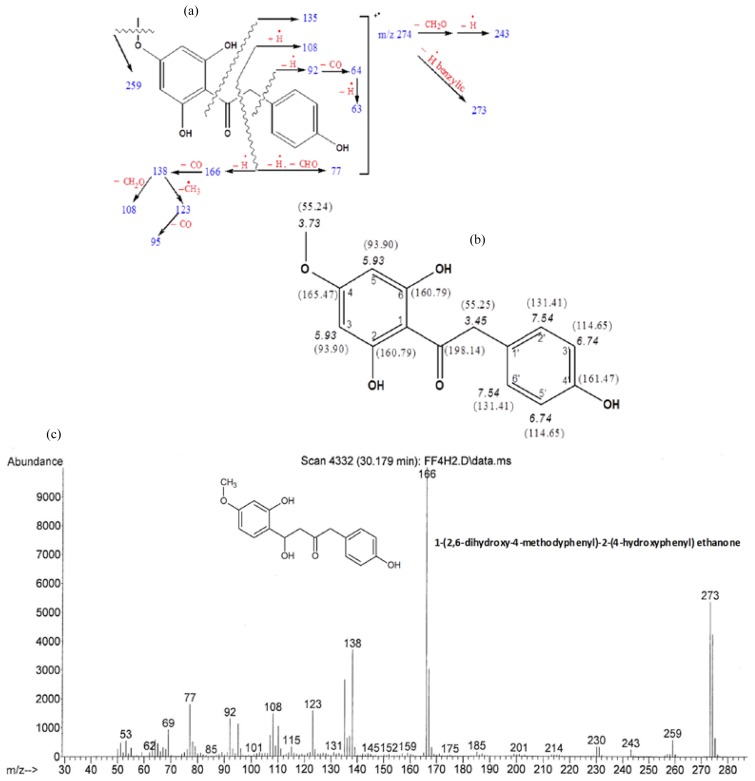
(**a**) Proposed fragmentation of DMHE; (**b**) structure of DMHE showing assignment of protons and carbons; and (**c**) mass spectrum of gas chromatography-mass spectrometry (GC-MS) analysis of DMHE.

DMHE isolated from fraction FF-3 eluted at a retention time 30.13 min GC-MS and displayed a molecular ion peak at *m*/*z* 274 in its mass spectrum. This was deduced as 1-(2,6-dihydroxy-4- methoxyphenyl)-2-(4-hydroxyphenyl) ethanone based on the fragment ion peaks of its mass spectrum as shown in Figure 2c. The justification for this assignment of structure is summarized as follows: in Figure 2a, the ion pairs primarily produced by the cleavage on either side of the carbonyl group can be observed at *m*/*z* 135, 166, 108 and 77. The M-1 peak at *m*/*z* 273 supports the presence of a benzyl moiety, where the easy loss of H by benzylic cleavage is a possible process. The peaks at *m*/*z* 108 and 77 on the one hand, and the peaks at *m*/*z* 92, 64, and 63 on the other, justify the presence of a *p*-hydroxybenzyl moiety on one side of the carbonyl group; while the peaks at *m*/*z* (molecular weight) 166, 138, 108, 123, and 95 justify the presence of a 2,6-dihydroxy-4-methoxyphenyl moiety on the other side of the carbonyl group. Justifications for the presence of a methoxy group are shown by appearance of prominent peaks at *m*/*z* 259 which is consistent with the loss of a methyl group from the parent ion.

In the NMR (nuclear magnetic resonance) spectrum, the two pairs of equivalent aromatic hydrogens, H_2′_/H_6′_ and H_3′_/H_5′_, in the *p*-disubstituted benzene and the pair of equivalent aromatic hydrogens in the 2,6-dihydroxy-4-methoxyphenyl group, H_3_/H_5_, can be seen at δ 7.54 (d, 8.6 Hz), 6.74 (d, 8.6 Hz), and 5.93 (s), respectively. The corresponding three equivalent pairs of aromatic carbons, C_2′_/C_6′_, C_3′_/C_5′_, and C_3_/C_5_, can also be seen at δ 131.41, 114.65, and 93.90 in the ^13^C NMR spectrum (CDCl_3_/CD_3_OD, 67.5 MHz). One aromatic methoxy group is also shown by the signals at δ 3.73 (−OCH_3_) and 55.24 (methyl C), respectively. In the ^1^H NMR spectrum, the signal for the two benzylic hydrogens must have fallen under the strong solvent (methanol) peak at δ 3.45. However, from the normal and DEPT ^13^C NMR, methylene carbon at δ 55.25 justifies one benzylic methylene group connected to a carbonyl group. The ketone carbonyl signal appears at δ 198.14. Together with the chemical shifts for the remaining carbons, the assignments of all the NMR signals are summarized in the structural Figure 2b.

### 2.3. Cytotoxicity Screening Using MTT Cell Proliferation Assay

**Figure 3.**
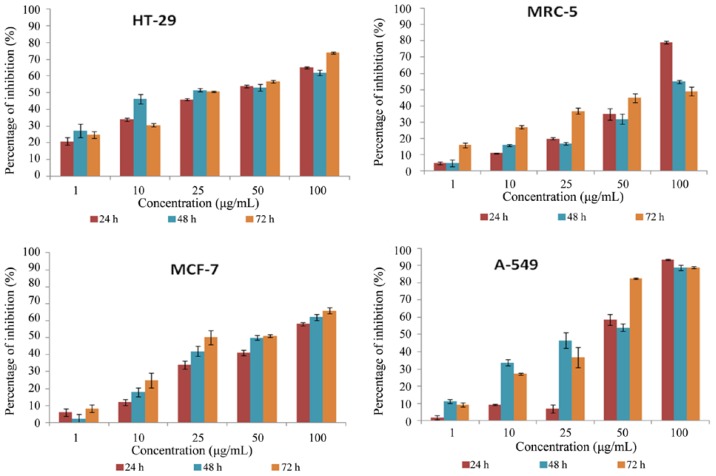
*In vitro*, cytotoxic effects of DMHE on HT-29 colon cancer cells, CaSki cervical cancer cells, MCF-7 breast cancer cells and MRC-5 normal lung cells. Cells were treated with various concentrations of DMHE from the ethyl acetate fraction of *Phaleria macrocarpa* (Scheff.) Boerl fruits for 24, 48 and 72 h prior to the determination of cytotoxicity by MTT cell proliferation assay. Each value is expressed as mean ± standard deviation of three measurements.

Cytotoxicity determination, a common method of evaluating the biological activity of natural products, is useful in confirming whether plant extracts have potential antiproliferative properties [9]. In our present study, HT-29, MCF-7, A549 and MRC-5 cells were treated with various concentrations (1, 10, 25, 50 and 100 μg/mL) of DMHE for 24, 48 and 72 h, respectively. DMHE exhibited high cytotoxic effect in HT-29 cells with IC_50_ values of 38.8 ± 1.64, 17.2 ± 2.29 and 25.3 ± 0.99 μg/mL at 24, 48 and 72 h, in MCF-7 cells with *IC*_50_ values of 80.1 ± 2.3, 48.3 ± 1.43 and 25.0 ± 2.65 μg/mL at 24, 48 and 72 h and in A549 with *IC*_50_ values of 45.0 ± 2.21, 37.5 ± 2.66 and 31.8 ± 1.69 at 24, 48 and 72 h respectively. In contrast, the proliferation of the normal human fibroblast breast cell line MRC-5 was only marginally affected by treatment with DMHE (*IC*_50_ values of 66.8 ± 1.19, 90.0 ± 1.53 and ≥100.0 ± 1.9 μg/mL for 24, 48 and 72 h respectively. This data is shown in Figure 3. Based on this data, we choose to further characterize the effects of DMHE on HT29 cells, which was the most susceptible cell line. Morphological changes of apoptotic bodies, quantification of early and late apoptotic cells, DNA content in cell cycle check point control and expression of protein were characterized.

### 2.4. Morphological Examination of Apoptosis

#### 2.4.1. Inverted and Phase Contrast Microscopic Examination

**Figure 4.**
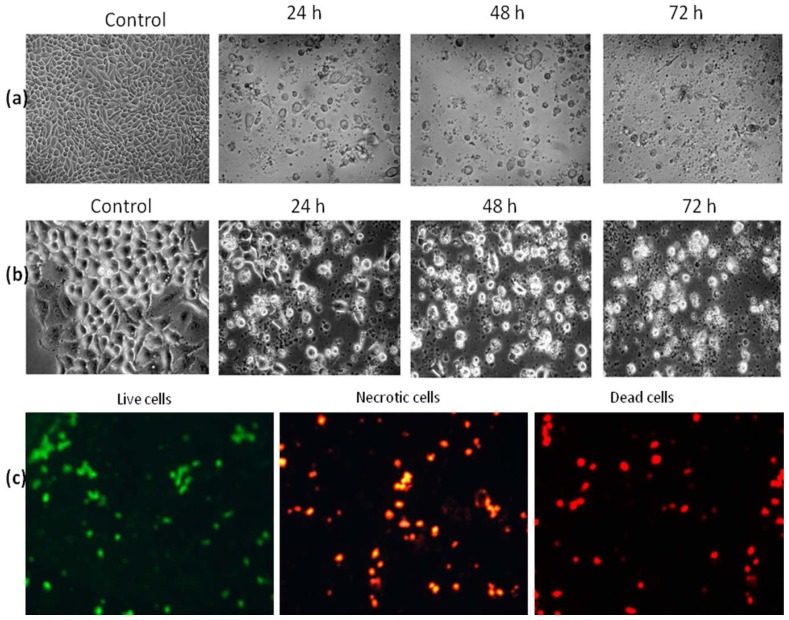
(**a**) Treatment with DMHE (38, 17 and 25 μg/mL) for 24, 48 and 72 h induces morphological changes in HT-29 colon cancer cells. Control or treated HT-29 cells as observed under inverted microscope and photographed; (**b**) control or treated HT-29 cells as observed under phase contrast microscope and photographed; and (**c**) treatment with DMHE for 48 h induces morphological changes in HT-29 colon cancer cells. After staining with acridine orange and propidium iodide, treated cells were observed under fluorescence microscopy—live cells stained (*green*), apoptotic cells (*orange*) and necrotic cells or dead cells (*red*).

Morphological changes in the HT-29 cell treated with 25 μg/mL of DMHE at 24, 48 and 72 h incubation were observed under an inverted and phase contrast microscope (Leica, Wetzlar, Germany). The cells indicated the highest effects after treatment with DMHE for 48 h. Microscopic observations revealed that more than 50% of the cells showed membrane blebbing, ballooning, chromatin condensation, and the formation of apoptotic bodies as shown in Figure 4a,b.

#### 2.4.2. Fluorescence Microscopic Examination

Morphological changes of individual HT-29 cells in the cell population were observed by fluorescence microscopy. The AO/PI (acridine orange and propidium iodide) procedure stains the DNA within the nuclei. Our results showed that viable cells displayed bright green nuclei, the early apoptotic cells exhibited orange nuclei, and the necrotic cells or dead cells displayed red nuclei as shown in Figure 4c. Thus, the morphological analysis of AO/PI stained HT-29 cells indicated significant morphological changes.

### 2.5. Annexin V Staining Assay

Apoptosis is a type of programmed cell death, used by multi-cellular organism to purge extraneous cells. It differs from necrosis in that it lasts a lifetime and is actually beneficial to the body, while necrosis is itself a form of cell death caused by acute cellular damage [10]. Double staining with annexin V-FITC (fluorescein-isothiocyanate) and a propidium iodide solution can distinguish between necrotic cells, live cells, early apoptotic cells and late apoptotic cells or dead cells. Flow cytometry results of double staining with Annexin V-FITC and propidium iodide solution can be interpreted as follows: the upper left quadrant (UL)—primary necrotic cells, the upper right (UR)—late apoptotic or secondary necrotic cells, the lower left quadrant (LL)—viable or live cells and the lower right quadrant (LR)—cells were undergoing apoptosis.

**Figure 5.**
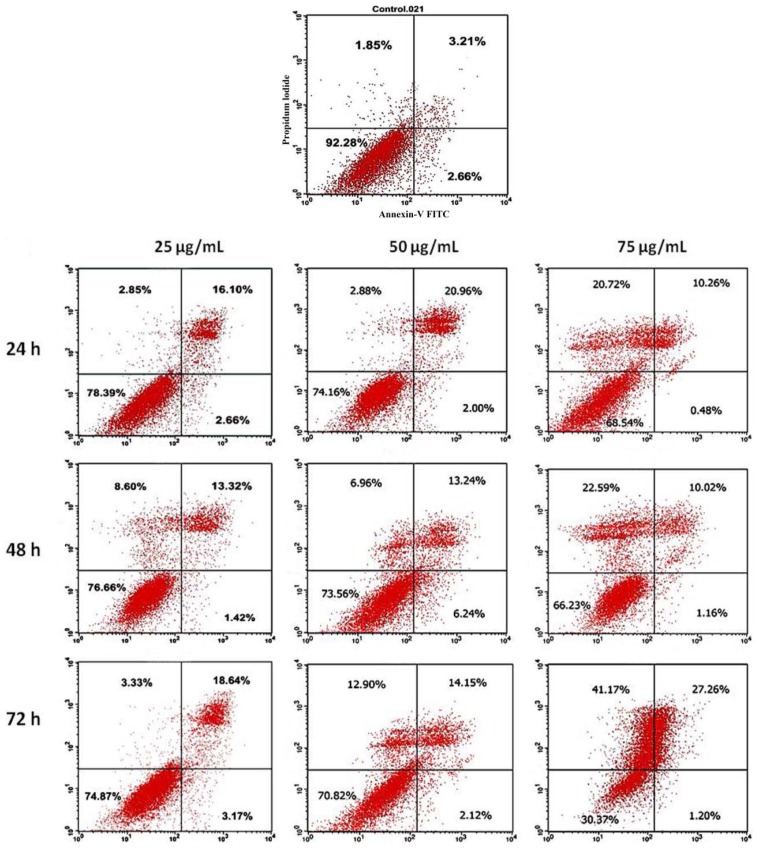
Effects of DMHE on induction of cell death in HT-29 cells. The cells were treated with different concentrations of DMHE (25, 50 and 75 μg/mL) in a time dependent manner (24, 48 and 72 h), labelled with FITC (fluorescein-isothiocyanate) annexin V and PI (propidium iodide).

Our results showed that for untreated HT-29 cells, 92.28% of the cells were viable, 2.66% in early apoptosis, 1.85% of the cells were necrotic cells and 3.21% of the cells were in late apoptosis or were dead cells. In HT-29 cells treated with 25, 50 and 75 μg/mL of DMHE, the percentage of viable cells decreased to 78.39%, 74.16% and 68.54%, the percentage of necrotic cells were 2.85%, 2.88% and 20.72%, early apoptotic cells decreased to 2.66%, 2.00% and 0.48%, and dead or late apoptotic cells were 16.10%, 20.96% and 10.26% respectively (for 24 h incubation). After 48 h incubation, the live cells decreased 76.66%, 73.60% and 66.23%, the necrotic cells were 8.60%, 6.96% and 22.59%, the early apoptotic cells were 1.42%, 6.24% and 1.16%, and the dead or late apoptotic cells decreased to 13.32%, 13.24% and 10.02%. After 72 h of incubation, the live cells decreased to 74.87%, 70.82% and 30.37%, the necrotic cells were 3.33%, 12.90% and 41.90%, the early apoptotic cells decreased to 3.17%, 2.12% and 1.02%, and the late apoptotic or dead cells were 18.64%, 14.15% and 27.26%. As shown in Figure 5a, the early apoptotic cells (LR) was almost absent. In this condition, it is difficult to ascertain whether the cells in UR are necrotic or late apoptotic cells.

**Figure 6.**
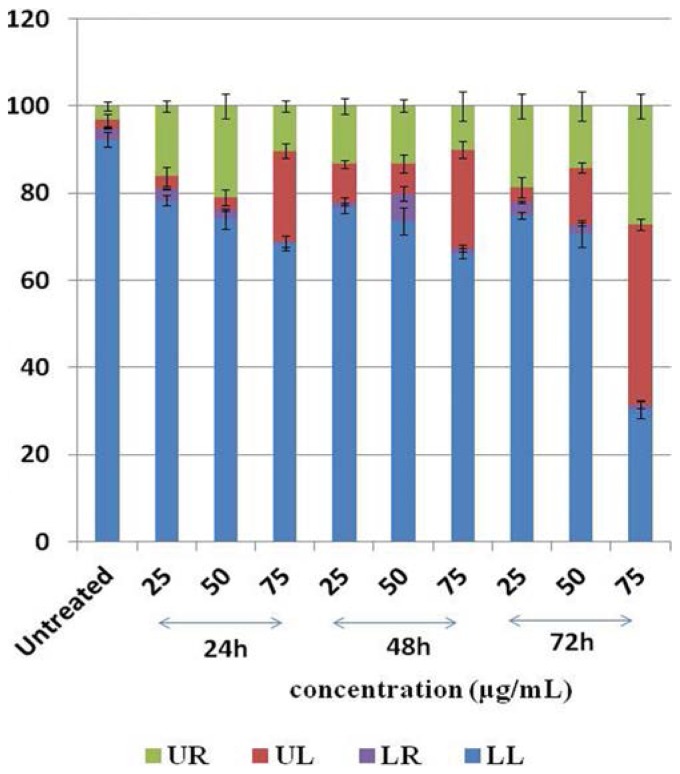
Histogram representation of the quantitative percentage of viable cells (LL), early apoptotic cells (LR), necrotic cells (UL) and late apoptotic cells (UR) of HT-29 treatment with different concentration of DMHE for 24, 48 and 72 h. Viable cells = LL; Early apoptotic cells = LR; Late apoptotic cells or dead cells = UR; Necrotic cells = UL.

Our results suggested that although apoptosis may be one of the mechanisms by which DMHE induces cell death, the primary mechanism involved may be necrosis. This effect was shown to be both dose and time dependent. All corresponding data are shown in Figures 5 and 6.

### 2.6. Cell Cycle Analysis

Cell cycle arrest and apoptosis are two important mechanisms involved in anti-cancer drug treatment [11,12]. The cell cycle plays an important role in cell fate, including cell replication, cell death, and cell function. The cell cycle consists of five stages: the first is the S phase, during which DNA replication occurs; the second is the G_1_ phase, which follows mitosis and during which the cell is sensitive to positive and negative cues from growth signaling networks; the third stage is the G_2_ phase, which is preceded by the S phase when the cell prepares for entry into mitosis; the next stage is mitosis or M phase, during which the cell divides into two daughter cells which have genetic material identical to each other and to the mother cell; and the final stage is the G_0_ phase, which is when cells have reversibly withdrawn from the cell division cycle in response to high cell density.

Terminal differentiation or senescent out-of-cycle states are also possible, resulting in cells that are irreversibly withdrawn from the cell cycle. The synthesis of DNA and DNA staining increases during the S phase. The G_2_ phase is when 4*n* DNA is present just before mitosis and the M phase is when 4*n* DNA is present during mitosis itself. These phases are indistinguishable from one another using just a DNA probe and a flow cytometer.

**Figure 7.**
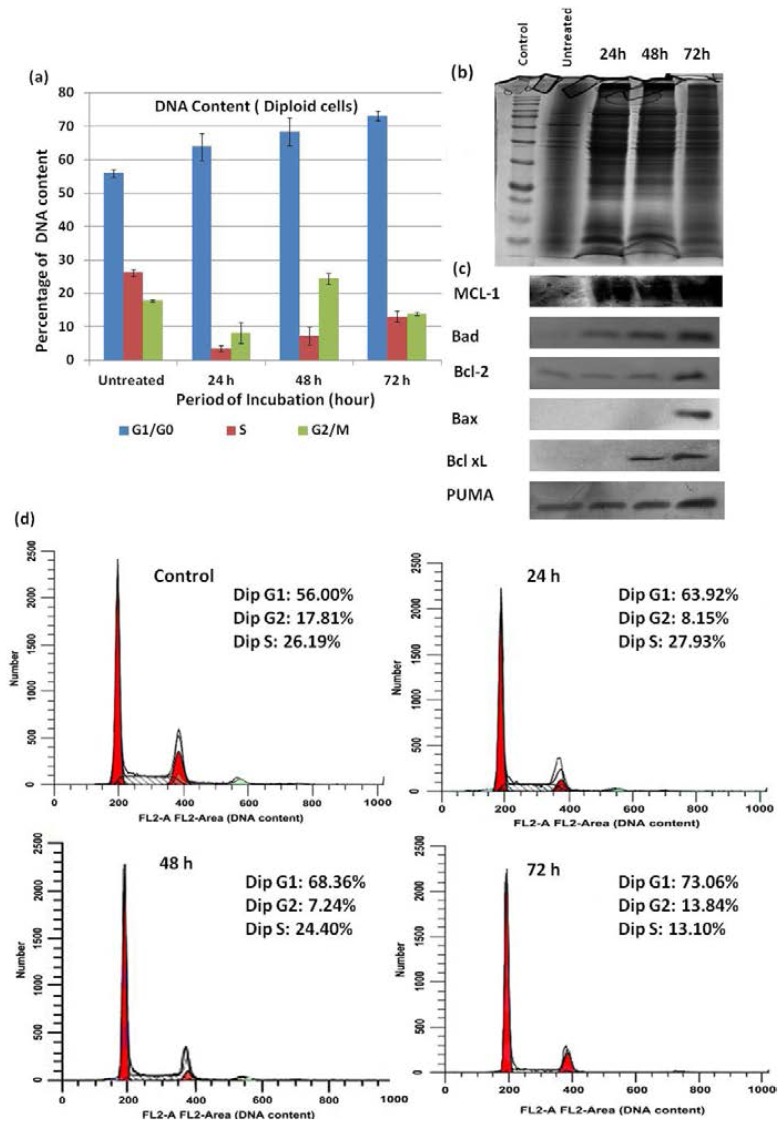
(**a**) Effect of DMHE on the HT-29 cell cycle. Cells treated with DMHE (25 μg/mL) for 24, 48 and 72 h, and analyzed by flow cytometry after staining with PI. Percentages of diploid cells (DNA content) at G_0_/G_1_, S, and G_2_/M phases of HT-29 cells were determined after 24, 48 and 72 h incubation periods; (**b**) coomasie blue stained SDS-PAGE gels demonstrating equivalent loading of proteins; (**c**) the expression of anti-apoptotic and pro-apoptotic proteins as detected by western blotting; and (**d**) histogram showing quantitative percentages of diploid cells or DNA content in each cell cycle phase without treatment and with treatment.

Our results showed that there was a statistically significant difference in the cell cycle phases between treated cells and untreated cells (negative control) in the G_0_/G_1_, S, and G_2_/S phases. Compared to the negative control, DMHE treatment for 24 h, 48 h and 72 h induced an acceptable arrest of HT-29 cells in the G_0_/G_1_ phase of the cell cycle. Following treatment with DMHE for various time periods (24, 48 and 72 h), the HT29 cells were stained with PI (propidium iodide). The distribution of the cells in the various phases of the cell cycle was then analyzed with a flow cytometer and the percentage of cells in the G_0_/G_1_ phase, the S phase and the G_2_/M phase were calculated using Modfit software. The results are presented in Figure 7.

Our results showed that after 24, 48 and 72 h of treatment with 25 μg/mL of DMHE, the percentage of cells in the G_0/_G_1_ phase significantly increased to 63.92%, 68.36% and 73.06%, respectively. The percentage of cells in the G_2_/M phase increased to 8.13%, 24.4% and 13.84% and the percentage of cells in the S phase also dramatically increased to 3.54%, 7.24% and 13.1%. When the cells were treated with 25 μg/mL of the compound, significant G_0_/G_1_ phase arrest was observed.

HT29 cells treated with DMHE showed significant cell cycle arrest in the G_0_/G_1_ phase in a time-dependent manner, compared to untreated cells. The standard deviation was calculated between the treated and untreated cells. In untreated cells, the percentage of DNA content was 56.00% at the G_0/_G_1_ phase, 26.19% at the S phase and 17.81% at the G_2_/M phase. Our results suggested that DMHE induced cell death in HT-29 cells. The G_0_/G_1_ arrest and inhibition of cell growth could be a result of the induction of necrosis or late apoptosis, which may be mediated by cell cycle arrest in the G_0_/G_1_ phase.

### 2.7. Western Blot Analysis

Apoptosis is a developmentally programmed form of cell death. It plays an essential role in both carcinogenesis and cancer treatment and is a primary target of many treatment strategies. Apoptosis eliminates excess damaged or unwanted cells and is a regulated pathway important for maintaining homeostasis in multi-cellular organisms. Apoptosis can be initiated by various external and internal signals and these signaling pathways are controlled by the regulated expression(s) of apoptosis-associated genes and protein. The Bcl-2 family of proteins (B cell lymphoma 2) plays an important role as anti-apoptotic and pro-apoptotic proteins. There are two groups of proteins involved in the regulation of apoptosis—anti-apoptotic or pro-survival proteins such as Bcl-2, Bcl-xL Bcl-w, Mcl-1 and A1 and pro-apoptotic proteins such as Bax, Bak, PUMA, Bok, Bad, Bid, Bik, Blk, Hrk, BNIP3 and BimL.

Bcl-2 proteins are alternative targets for both pro- and anti-apoptotic therapeutic approaches [13]. To elucidate the possible mechanism(s) by which DMHE induced death of HT-29 cells, the cells were incubated at *IC*_50_ concentration (25 μg/mL) of DMHE for 24, 48 and 72 h. The change in expression of the Bcl-2 family of proteins that may be involved in compound–induced cell death was then observed using western blotting. It was found that upon treatment with DMHE, the expression of the pro-apoptotic protein Bax, was up-regulated in a time-dependent manner. In contrast, the pro-apoptotic protein Bak, was not up-regulated. The anti-apoptotic proteins Mcl-1, PUMA, Bcl-2 and Bcl-xL were also up-regulated in a time-dependent manner as shown in Figure 6c,d. Treatment with DMHE also caused the up-regulation of Bcl-2 in a time-dependent manner. These results suggested that the G_0_/G_1_ arrest and, consequently, the apoptosis inducing effect of DMHE on HT-29 cells may be associated with the up-regulation of the Bcl-2 family of proteins.

## 3. Experimental Section

### 3.1. Isolation of Bioactive Compound

*P. macrocarpa* fruits were purchased from Yogyakarta, Indonesia since July 2011. The fruits (~1000 g) were exhaustively soaked in 80% aqueous methanol for three days at room temperature. Dry methanol extracts were obtained after removing the solvent by evaporation under reduced pressure to produce a dark brown crude methanol extract (47.2 g, 4.7%). The methanol extract (47.2 g) was further extracted with hexane to produce a hexane-soluble fraction (2.10 g, 0.20%). The hexane-insoluble residue was then partitioned between ethyl acetate and distilled water (1:1, 500 mL:500 mL) to give an ethyl acetate soluble fraction (12.2 g, 1.22%) and a water-soluble fraction by freeze-drying (29.8 g, 2.90%). The weight of the methanol extract and the fractions were determined after solvent evaporation. The bioactive dried ethyl acetate extract was then separated on a silica gel column (Merck, Darmstadt, Germany). Elution was performed using a solvent mixture of hexane/ethyl acetate with an increasing volume of ethyl acetate (100:1 to 1:100). Monitoring was performed using thin layer chromatography (TLC). The successive fractions were then collected and dried under vacuum using a rotary evaporator. the resulting fractions were screened by TLC. Stock solutions were prepared at a concentration of 10 mg/mL. The final concentrations of test samples were 10, 25, 50 and 100 μg/mL.

### 3.2. GC-MS (Gas Chromatography-Mass Spectrophotometry) and NMR (Nuclear Magnetic Resonance) Analysis of Bioactive Compound

GC-MS analysis was based on the protocol described by Sri Nurestri Malek [14] and NMR analysis was carried out on a JEOL 400 MHz FT-NMR, (JEOL, Tokyo, Japan) with TMS as internal standard, ^1^H NMR (400 MHz), and ^13^C NMR (400 MHz) spectra were obtained.

### 3.3. Cytotoxicity Screening

#### 3.3.1. Cell Culture

The HT-29 human colon adenocarcinoma cells, MCF-7 human fibroblast breast cancer cells, A-549 human lung cancer cells and MRC-5 normal human fibroblast lung cells were purchased from the American Type Culture Collection (ATCC, Boulevard Manassas, VA, USA). The cells were cultured and maintained in RPMI 1640 (Sigma-Aldrich, St. Louis, MO, USA) medium for HT-29, MCF-7 and A549 cells and Eagle’s Minimum Essential Medium (EMEM, Sigma) for the MRC-5 cells. They were supplemented with 10% fetal bovine serum, 100 μg/mL penicillin/streptomycin, and 100 μg/mL of amphotericin B and the cells were incubated at 37 °C in a humidified atmosphere containing 5% CO_2_.

#### 3.3.2. MTT Cell Proliferation Assay

The cytotoxic effect of DMHE on selected cancer cell lines and normal cell line were determined using the MTT cell proliferation assay. The trypan blue exclusion assay was used to analyze cell viability [15,16]. In brief, the process involved spinning of cells from a confluent tissue culture flask at 1000 rpm for 5 min and re-suspending them with 1.0 mL of growth medium. The cells were then cultured in 96 well plates and incubated in a CO_2_ incubator at 37 °C for 24 h. After 24 h, the medium was removed and DMHE at varying concentrations (1, 10, 25, 50 and 100 μg/mL with 200 μL of 10% medium) were added into each well. For negative control, cells were not treated with DMHE. After incubating for 24, 48 and 72 h, each well was added with 10 μL MTT stock and incubated in a dark place for three hours. The medium with the MTT solution was then removed and 200 μL of absolute DMSO (Dimethyl sulfoxide) was added to each well in order to solublilize formazan crystals that had been formed. The resulting amount of formazan was measured at 540 nm using an ELISA plate reader. The assay was performed in triplicate.

The percentage of inhibition (%) was calculated according to the following formula:

(1) $$\text{Percentage of inhibition }(\%)=\frac{\text{OD control}-\text{OD sample}\times 100\%}{\text{OD control}}$$

where OD = optical densties, control = without treatment with compound, sample = with treatment with compound.

The *IC*_50_ value was determined as the concentration of test compounds that caused 50% inhibition or cell death (average from three experiments). The *IC*_50_ values of the test compounds were obtained by plotting the percentage of inhibition *versus* the concentration of test compounds as previously described [14,17–19].

### 3.4. Morphological Examination of Apoptosis

#### 3.4.1. Inverted and Phase-Contrast Microscopy

The HT-29 cells were plated in petri dishes (30 mm) at a density of 1 × 10^5^ cells/well. DMHE was then added to each well and incubated at 37 °C in humidified 5% CO_2_ for 24, 48 and 72 h. After the various incubation periods, the petri dishes were examined under an inverted and a phase microscope (Leica).

#### 3.4.2. Fluorescence Microscopy

Acridine orange and propidium iodide (AO/PI) double staining method was used to observe apoptotic morphological changes in each cell body under fluorescence microscopy (Leica). The HT-29 cells were seeded onto a petri dish (30 mm) at a density of 1 × 10^5^ cells/mL. The cells were then treated with DMHE and incubated in a CO_2_ incubator for 48 and 72 h. After the incubation period, the cells were detached with an accutase solution, centrifuged at 1000 rpm for 5 min and washed twice with PBS, after which the supernatant was discarded and the cells re-suspended in 1 mL of PBS. 100 μL of cells were incubated with 5 μL of acridine orange (AO) and 5 μL of propidium iodide (PI) for 10 min at room temperature under dark conditions. Stained cells (10 μL) were placed onto coated glass slides and 20 μL of mounting media and stored at −20 °C until analysis. The stained cells were then photographed under a fluorescence microscope (Leica).

### 3.5. Annexin V Staining Assay

Approximately 1 × 10^6^ HT-29 cells were incubated in petri dishes for 24 h. Various concentrations of DMHE (25, 50 and 75 μg/mL) were then added to the cells and incubated for 24, 48 and 72 h, respectively. The cells were harvested with accutase in phosphate buffered saline by centrifugation, stained with annexin V-FITC only, propidium iodide only and double stained with annexin V-FITC/PI according to the Annexin V-FITC Apoptosis Detection Kit protocol (BD Pharmingen™, BD Bioscience, San Jose, CA, USA). The stained cells were examined using a FACScalibur flow cytometer (BD Bioscience) and the data analyzed using CellQuest Pro analysis software (Becton Dickinson, San Jose, CA, USA). A total of 10,000 cells were counted for each treatment, and the apoptotic and total death rates were determined.

### 3.6. Cell Cycle Analysis

HT-29 cells (1 × 10^5^) were cultured in petri dishes and incubated for 24 h. DMHE (25 μg/mL) was then added and the cells were incubated for 24, 48 and 72 h at 37 °C in a 5% CO_2_ incubator. After the various incubation periods, the cells were harvested with accutase, centrifuged at 1000 rpm for 5 min, and washed with warm PBS. The pellets were re-suspended in 300 μL of PBS and was fixed by adding 700 μL of cold absolute ethanol and incubated at 20 °C overnight. The next day, the cells were centrifuged at 1000 rpm for 5 min and the supernatant was removed. The cells were then washed with cold PBS twice and stained with propidium iodide using the Cycle TEST™ PLUS DNA Reagent Kit (Becton Dickinson). The cells were sorted in a FACScalibur flow cytometer (BD Bioscience) using CellQuest pro software (Becton Dickinson) and a quantitative analysis of the cell cycle distribution was carried out using a trial version of the ModFit LT V4.0 software (Verity Software House, Topsham, ME, USA). The significance of the differences was determined using the Student’s *t*-test.

### 3.7. Western Blot Analysis

The HT-29 cells were treated with DMHE at 25 μg/mL for 24, 48 and 72 h respectively. After incubation, the cells were harvested with accutase and washed with cold PBS three times. The cells were then lysed in a protein lysis buffer containing protease inhibitors. The lysates were centrifuged at 13,000× *g* for 15 min at 4 °C and the supernatant was collected and kept at 4 °C until the SDS-PAGE analysis (Bio-RAD, Hercules, CA, USA). The Protein concentration was determined using the BCA protein assay kit (Invitrogen, Grand Island, NY, USA) with bovine serum albumin as the standard.

Extracted proteins (20 mg/mL) were separated on 12% sodium dodecyl sulfate-polyacrylamide gels (SDS-PAGE). Electrophoresis was carried out at 20 V/cm for three hours. The proteins in the SDS-PAGE electrophoresis gels were transferred onto polyvinylidene difluoride (PVDF) membranes by semi-dry transfer (Membrane Solution, North Bend, OH, USA). The membranes were washed, blocked and incubated with primary antibodies for Bcl_2_, Bcl-xL, PUMA, Mcl-1, Bak, Bad, Bax. They were then washed and incubated with the respective secondary antibodies at room temperature. Western blot analysis was then performed according to the Western Max™ Horseradish Peroxidase Chromogenic Detection Kits protocol (AMRESCO, Solon, OH, USA). The resulting membranes were dried, photographed and stored in the dark.

### 3.8. Data Analysis

A hemocytometer was used to determine cell numbers and trypan blue exclusion was used to determine cell viability. All tests were performed at least three times. The data from the cytotoxicity and annexin V staining assay were expressed as mean ± standard derivations (SD). The mean and standard deviations in each phase of the cell cycle were calculated using Microsoft Excel software (Microsoft, Redmond, WA, USA). Statistical significance of results were determined by Student’s *t*-test and a *p* value of more than 0.05 was considered to be statistically significant.

## 4. Conclusions

The compound 1-(2,6-dihydroxy-4-methoxyphenyl)-2-(4-hydroxyphenyl) ethanone (DMHE) was isolated from the ethyl acetate fraction of the *Phaleria macrocrapa* (Scheff.) Boerl fruit using column chromatography. Its identity was confirmed by gas chromatography-mass spectrometry (GC-MS) analysis and NMR. DMHE was found to cause a significant decrease in cell proliferation in HT-29 cells in a dose- and time-dependent manner. Morphological features of apoptosis such as cell death, cell shrinkage, membrane blebbing, nuclear condensation and fragmentation were observed under inverted, phase and fluorescence microscopy. Western blot revealed an up-regulation of the pro-apoptotic protein Bax. However, the anti-apoptotic proteins Mcl-1, PUMA, Bcl-2 and Bcl-xL were also found to be increased in a time-dependent manner. The expression of the pro-apoptotic protein Bak was however, not increased. Results from western blot analysis somewhat agreed with observations made in the Annexin V-FITC/PI flow cytometry analysis. The flow cytometry analysis suggested that a smaller percentage of cells were in early apoptotis following treatment with DMHE. Most of the cells were observed to be in primary necrotic, secondary necrotic or late apoptotic state after treatment with DMHE. However, it must be noted that it was not possible to distinguish between necrotic, late apoptotic and dead cells using the Annexin V-FITC/PI assay. Therefore, cells may have died either by necrosis or apoptosis, caused by tissue types, stage of development, physical disruption, cell death signal’s nature and physiological environment; further experiments are needed to confirm these observations. However, DMHE did display potent anti-cancer activity by selectively inducing death of cancerous cells.
